# Immunotherapy With Egg Yolk *Eimeria* sp.-Specific Immunoglobulins in SPF Leghorn Chicks Elicits Successful Protection Against *Eimeria tenella* Infection

**DOI:** 10.3389/fvets.2021.758379

**Published:** 2021-11-11

**Authors:** Marco A. Juárez-Estrada, Guillermo Tellez-Isaias, Félix D. Sánchez-Godoy, Rogelio A. Alonso-Morales

**Affiliations:** ^1^Departamento de Medicina y Zootecnia de Aves, FMVZ, Universidad Nacional Autónoma de México, Mexico City, Mexico; ^2^Departamento de Genética y Bioestadística, FMVZ, Universidad Nacional Autónoma de México, Mexico City, Mexico; ^3^Department of Poultry Science, University of Arkansas, Fayetteville, AR, United States

**Keywords:** immunoglobulin Y, coccidiosis, apicomplexa, chickens, passive immunity, prepatency, gut health, dysbiosis

## Abstract

Avian coccidiosis is the first to most economically important parasite disease affecting poultry industries worldwide. Current prevention measures are largely based upon prophylactic chemotherapy supplemented by the application of live attenuated or wild-type parasite vaccines. However, the rising appearance of drug resistance, consumer's concern for antibiotics use in poultry production and higher manufacturing cost of live vaccines has driven to adopt new technologies aimed at increasing animal health and production efficiency. Supplementing chickens with egg yolk *Eimeria* sp.-specific immunoglobulins can be a viable alternative to avoid severe outbreaks of the disease. Twelve-week-old SPF White Leghorn chickens were experimentally infected with a large dose of *E. tenella*. During the prepatent period, the birds were supplemented by oral gavage with 60 or 120 mg/bird of hyperimmune egg yolk *Eimeria* species-specific immunoglobulins Y (Supracox®, SC) on a daily basis. The animals were euthanized 7 days post-infection (PI) and their passive immune protection was evaluated. Birds treated with 120 mg/bird of SC showed more viability, increased body weight gain (BWG), a normal hematocrit level (HCT), reduced oocyst output per gram of feces (OPG) or cecal tissue (OPGC), and fewer cecal lesions compared to the untreated infected (UI) control group. Birds supplemented with 60 mg/bird of SC did not show any significant difference on BWG, HCT, OPG, OPGC, and cecal lesion score when compared with the UI group. An ELISA test of the SC showed a weak cross-reactivity of IgY toward two asexual zoite stages of *E. tenella*. Western blot analysis of the sporozoite with SC showed few antigens barely recognized, while more stained bands were detected in the merozoite (≈82, ≈60, ≈54, ≈40, ≈38, ≈27.5, and ≈13 kDa). Oral immunotherapy using egg yolk polyclonal IgYs against *Eimeria* sp. represents an effective and natural resource against severe *E. tenella* infection favoring the gradual withdrawal of the anticoccidial drugs and antibiotics.

## Introduction

Avian coccidiosis is one of the most common enteric diseases of poultry, and it is caused by several protozoan parasites of the genus *Eimeria* (1). The disease is characterized by reduced weight gain and feed conversion efficiency and is the most economically significant parasitic infection of the global poultry industry (2). *Eimeria tenella* is one of the seven most prevalent *Eimeria* species in chickens; it is the causative agent of cecal coccidiosis, causing severe tissue damage that results in hemorrhage, nutrient malabsorption, diarrhea, and gut microecological disturbance (3, 4). Coccidiosis is controlled prophylactically with anticoccidial feed drugs (2). Vaccination with live or attenuated parasites is routinely used to confer protection by adaptive immunity (5). Nevertheless, both measures are associated with some drawbacks, including the emergence of drug resistance and the potential reversion to virulence of the live vaccines, on top of their high production expenses (2). These issues have driven the development of new control strategies (6, 7). Several anticoccidial products have been tested as potential alternatives for drug or live vaccines; these include a sub-unitary gametocyte vaccine (8–11), herbal extracts (12), probiotics (13), and antibodies (10, 11, 14–17).

Humoral immunity is the primary defense to eliminate toxins or invading pathogens (18, 19). Passive immunization relies on the transfer of humoral immunity in the form of active antibodies from one individual to another (15, 20). Antibodies are regularly obtained from mammalian or chicken sera; however, this procedure is invasive and animals usually have to be euthanized in order to obtain a sufficient amount of antibodies (6, 18). The isotype antibody transferred from the serum of breeder hens to egg yolks is immunoglobulin Y (IgY), which offers natural passive immunity to offspring (9, 11, 15). Various studies have demonstrated that the immunization of breeder hens and passive transmission to their offspring can play important roles in combatting coccidiosis (8, 9, 15). However, few studies have been conducted to evaluate the protection of poultry from coccidiosis by passive immunization using egg yolk pathogen-specific secretory hyperimmune antibodies as immunotherapy (6, 18). Oral passive immunoglobulin Y-based immunotherapy is a highly attractive strategy in that it is environmentally friendly, nontoxic, and reduces the numbers of animals required for antibody production (6). Lately, it has received special attention because of the feasibility of large-scale commercial production, the non-invasive methods employed, and its cost-effective features (6).

The prolonged use and misuse of conventional antimicrobial drugs has spawned antibiotic resistant microbes prompting scientists to search for other microbial-control options. In particular, the use of IgY as a novel mode of immunotherapy using oral chicken immunoglobulin Y to confer passive immunity has gained much interest as an inexpensive non-antibiotic alternative for the prophylaxis and treatment of a wide variety of infectious diseases (19). Several researchers have successfully tested the ability of egg yolk immunoglobulins to control infectious diseases in chickens (6, 18). In one study using egg yolk hyperimmune IgY powder obtained by spray-drying, Lee et al. (21) demonstrated that the continuous oral feeding of young chicks with egg yolk IgY powder from hens previously immunized against three species of *Eimeria* conferred significant protection against *E. acervulina* infection. Another study pointed out the efficacy of this same hyperimmune egg yolk IgY powder against infection with *E. maxima* and *E. tenella* (22). In a recent study, lyophilized egg yolk IgY powder from hens immunized against five *Eimeria* species was prophylactically given to chickens; it was demonstrated to be able to provide partial protection against experimental infection with *E. tenella* (23). However, there are no details about oral immune passive therapy using IgY antibodies against three different *Eimeria* species in birds infected with a large dose of a heterologous wild-type strain of *E. tenella*.

## Materials and Methods

### Animals

Sixty-four fertile specific-pathogen-free (SPF) eggs (provided by ALPES, S.A. de C.V. Tehuacan, Puebla, Mexico) were hatched in the Department of Avian Medicine and Poultry Science, College of Veterinary Medicine and Zootechnics (FMVZ), National Autonomous University of Mexico (UNAM). After hatching, all the 1-day-old SPF Leghorn chickens were reared in *Eimeria*-free floor pens inside a well-ventilated room. An appropriate light schedule, clean water, and feed without anti-coccidia drug were provided according to the life stage and strain of the chickens used.

### Parasites

The wild-type strain of *E. tenella* used here was isolated from a clinical outbreak in broilers housed in a farm in Queretaro, a central state in Mexico. The methodology used for the oocyst separation from feces, sporulation, and the storing of oocysts of *E. tenella* has been described elsewhere (24, 25). The procedures used for preparing infective doses and quantifying oocysts in feces have previously been described (25).

### Antisera and Egg Yolk Polyclonal IgYs Against *E. tenella*

The immune sera for the ELISA and Western blot analysis were obtained from six 8-week-old hybrid pullets (Hampshire × Rhode Island Red × Plymouth Rock Barred) inoculated by gavage with 5 × 10^3^ sporulated oocysts of *E. tenella*. All birds were boosted twice at 4-week intervals. The pullets were housed in cages with raised wire netting floor inside a coccidia-free room to avoid oocyst cross-contamination. The negative control birds (*n* = 4) were maintained under the same conditions in an adjacent room and were inoculated by gavage with phosphate-buffered saline solution (PBS; 0.01 M, pH 7.4) only. In order to obtain reference antisera, 2 weeks after the last immunization, all pullets were wing-bled. The blood was allowed to clot for 1 h at room temperature and then overnight at 4°C; afterwards, it was centrifuged at 2000 × g for 5 min, aliquoted, and stored at −20°C until use.

The chicken egg yolk polyclonal immunoglobulins against *E. tenella* were obtained from four SPF White Leghorn pullets (12 weeks old) injected subcutaneously with an experimental vaccine formulated with 5.3 × 10^6^ whole sporozoites of *E. tenella* mixed with a nanoparticle-based adjuvant (IMS 1313 N VG PR Seppic Montanide™, France) (EtSz-IMS1313) (26). All birds were boosted three times at 2-week intervals. The pullets were housed in cages with a raised wire netting floor inside a coccidia-free room. Negative control birds (*n* = 2) were maintained under the same condition in an adjacent room and were injected subcutaneously with PBS only. The reference antisera against the complete sporozoite of *E. tenella* were obtained from blood of every SPF White Leghorn chickens, it was collected from *Vena cutanea ulnaris* 1 week after the last immunization. Serum was harvested by clotting (1 h at room temperature and then overnight at 4°C, then it was centrifuged at 2000 × g for 5 min), aliquoted, and stored at −20°C until use. Two weeks after the last immunization some eggs from each SPF White Leghorn hens were picked up to obtain IgYs from the egg yolk.

### Egg Yolk Polyclonal IgY Antibodies for Immunotherapy Trial

A suspension of egg yolk hyperimmune IgY antibodies against *Eimeria* sp. (Supracox®, SC; Investigacion Aplicada, S.A, de C.V. IASA, Puebla, Mexico) was used in this study. According to the manufacturer, SC was prepared with egg yolks of specific pathogen-free Leghorn hens hyperimmunized with live oocysts of the three major *Eimeria* species, *E. tenella, E. acervulina*, and *E. maxima*. The immunization schedule of SPF White Leghorn hens was carried out by infecting birds with 6 × 10^3^ sporulated oocysts of *E. tenella, E. acervulina*, and *E. maxima* through the oral (natural) route. This immunization schedule started at 60 days of age and continued at 4-week intervals throughout the whole egg production cycle. IgY antibodies were obtained by removing the lipid components from egg yolk with solvents, followed by protein precipitation and purification (Avid AL, Unisyn Technologies, Tustin, CA, USA). Then, the IgYs were spray-dried and resuspended in sterile water. The protein concentration was evaluated with the Bradford reagent (BioRad, Hercules, CA, USA) following the manufacturer's instructions.

### Egg Yolk Polyclonal IgY Antibodies for Analysis

The egg yolk polyclonal IgYs against whole sporozoites of *E. tenella* were isolated according to the water solution method described by Akita and Nakai (27). Briefly, the egg yolk was separated from the white. The yolk was thoroughly mixed with fresh cold distilled water (1:10, *v/v*). The mixture was adjusted to pH 5.1 with 0.1 mol/L HCl, then incubated overnight at 4°C. The water supernatant containing the IgYs was collected by centrifugation at 10,000 × g for 25 min at 4°C, then filtered through Whatman no. 1 filter paper. Two-step salt precipitation was performed to purify the IgY in the filtrate supernatant. First, ammonium sulfate was added to a 35% saturation and the mixture was stirred at 4°C for 1 h. The precipitate was collected by centrifugation at 10,000 × *g* for 25 min at 4°C and suspended in deionized water (~1,500 μl). Secondly, the ammonium sulfate was added to a 20% saturation, and the mixture was stirred at 4°C for 1 h. The precipitate was again collected by centrifugation at 10,000 × *g* at 4°C for another 25 min and suspended in PBS. Finally, the precipitate was dialyzed against PBS. Gentamicin was added to the IgY preparation and adjusted at final volume of 1,200 μl (30 mg/ml) into 1.5 ml Eppendorf tube, and then it was frozen at −20°C until further analysis.

### Isolation and Purification of Two Asexual Zoite Stages of *E. tenella* (Sporozoites and Merozoites)

The release of sporocysts was achieved by vortexing sterilized sporulated oocysts (2.5 × 10^7^) at 2,000 rpm with 1 mm diameter glass beads (Sigma, St. Louis, MO, USA) for 1 min 10 sec. Sporocysts were purified using a 50% Percoll gradient (density 1.13 g/ml, GE Healthcare, Piscataway, NJ, USA) (28), resuspended at 1 × 10^6^/ml in excystation medium, and incubated at 42°C for 150 min with end-over-end mixing. The excystation medium consisted of PBS with 0.75% (*w/v*) taurodeoxycholic acid (Sigma, St. Louis, MO, USA) and 0.25% (*w/v*) trypsin from porcine pancreas Type II-S (Sigma, St. Louis, MO, USA). Freshly excysted sporozoites (Sz) were washed and then purified at a 60% Percoll gradient (density, 1.13 g/ml; GE Healthcare, Piscataway, NJ, USA) (28). Sporozoites for the immunization schedule were resuspended in sterile PBS divided into 1 mL aliquots, gradually frozen at −70°C at the rate of 1°C min^−1^ and stored at −70°C until required.

To obtain second-generation merozoites (Mz), three 10-week-old hybrid pullets were inoculated by gavage with 5 × 10^5^ sporulated oocysts of *E. tenella* and euthanized 112 h post-infection (PI). The intestines of these birds were processed for merozoite isolation, as described before by Liu et al. (29). The merozoites were purified using the method described by Geysen et al. (30). Each purified parasite (Sz and Mz) was suspended in sterile PBS with a protease inhibitor (cOmplete™ Roche Applied Science, Mannheim, Germany).

### Preparation of Antigens From Sporozoites and Merozoites

Both asexual zoites stages were separately disrupted by five freeze–thaw cycles. The final suspension was centrifugated at 2,000 × g for 18 min at 4°C. The supernatants were collected, and the protein concentration was assessed with the Bradford reagent (BioRad, Hercules, CA, USA), using a standard curve prepared with bovine serum albumin (Sigma, St. Louis, MO, USA) in the concentration range of 1–5 μg/μl. Antigens were stored in 200 μl aliquots at −70°C until they were required.

### SDS-PAGE and Antibody Purity Analysis of Egg Yolk IgYs From Supracox®

The quality and purity of immunoglobulins Y from SC were identified by sodium dodecylsulfate polyacrylamide gel electrophoresis (SDS-PAGE). SDS-PAGE was conducted under reducing and denaturing conditions with 7% stacking gel and 12% separation gel on an electrophoresis device (Bio-Rad Laboratories, Hercules, CA, USA). The protein bands were stained with Coomassie brilliant blue (CBB) (Bio-safe Coomassie, Bio-Rad, Hercules, CA, USA). The gel was analyzed measuring density after background subtraction using Image J.JS ImJoy gel analysis tool (http://rsbweb.nih.gov/ij/index.html). IgY antibody activity was evaluated with ELISA.

### ELISA

The reactivity of the anti-sporozoite serum and antisera against *E. tenella* to the sporozoite and merozoite antigens was evaluated by ELISA, essentially as described by Constantinoiu et al. (31). Briefly, 96-well microtiter plates (MaxiSorb, Nunc, Roskilde, Denmark) were coated at 4°C overnight with 1 μg of a sporozoite or merozoite antigen in 100 μl of carbonate buffer (0.1 M sodium bicarbonate and 0.1 M sodium carbonate, pH 9.6). Control wells were incubated with 100 μl of carbonate buffer only. After washing four times on a shaker with a saline solution (S) (120 mM NaCl, 25 mM Tris-HCl, pH 7.9) containing 1% Tween 20 (ST), non-specific binding sites were blocked by incubation with 110 μl of 5% skim milk in ST (STM) for 1 h at 37°C in a static oven. After four washes with ST, sera diluted in STM (1:10 and 1:100) were added to the test and control wells and incubated for 1 h at 37°C. Negative control sera from unimmunized birds were included in each plate. After incubation, the plates were washed four times with ST and incubated with their respective secondary antibody peroxidase conjugate (anti-chicken) (Jackson ImmunoResearch Laboratories, Inc. West Grove, PA, USA) diluted 1:1000 with STM. After 1 h of incubation at 37°C, the plates were washed four times and the enzymatic reaction was developed by adding 100 μl of OPD chromogen (*o*-phenylenediamine dihydrochloride, SIGMA, St. Louis, MO, USA) at a concentration of 5 μg/10 ml in citrate buffer (0.1 M citric acid, 0.1 M sodium citrate pH 4.5, and 20 μl of 30% hydrogen peroxide) for 10 min in a shaker under dark conditions. The absorbance produced by substrate hydrolysis was read at 450 nm with an ELISA microplate spectrophotometer (Epoch, BioTek, Winooski, VT, USA). All sera samples were analyzed in duplicate.

The IgYs in SC and egg yolk polyclonal immunoglobulins against the whole sporozoites of *E. tenella* were diluted (1:100) and detected with the secondary antibody peroxidase conjugate (anti-chicken) (Jackson ImmunoResearch Laboratories, Inc. West Grove, PA). When required, positive and negative controls sera from our SPF White Leghorn chicks immunization program were included at 1:100 dilution. The wells were filled with 100 μl of secondary antibody diluted 1:6,000 in STM. After 1 h of incubation at 37°C, the plates were washed four times and the enzymatic reaction was developed by adding 100 μl of the TMB chromogen (3,3′,5,5′-tetramethylbenzidine liquid substrate system, Sigma-Aldrich, St. Louis, MO, USA) at a concentration of 10 μg/10 ml in citrate buffer (0.1 M citric acid, 0.1 M sodium citrate pH 4.5, and 60 μl of 30% hydrogen peroxide) for 10 min in a shaker under dark conditions. The enzymatic reaction was stopped by adding 100 μl of 2N H_2_SO_4_ solution. The absorbance produced by substrate hydrolysis was read at 450 nm. All sera were analyzed in duplicate. All samples were analyzed three independent times to identify outliers.

### SDS-PAGE and Western Blot

Amounts of 20 μg of each purified fraction of sporozoites and second-generation merozoites were separated by 12% SDS-PAGE under reducing conditions. The resolved proteins were either stained by CBB or electrotransferred to polyvinylidene difluoride membranes (PVDF) (Bio-Rad, Hercules, CA, USA) according to Constantinoiu et al. (32). PVDF membranes were probed with anti-*E. tenella* sera (1:100), sera from uninfected birds (1:100), egg yolk IgYs from the SC (1:25), and egg yolk polyclonal specific anti-sporozoite antibodies (1:25). Horseradish peroxidase (HRP)-conjugated rabbit anti-chicken IgY (Jackson ImmunoResearch Laboratories, Philadelphia, PA, USA) were used as a secondary antibody at a dilution of 1:1500 (*v/v*) in 5% (*w/v*) STM. The blots were visualized with 3,3'-diaminobenzidine (10 mg/5 ml) (DAB tablets, SigmaFast™, Sigma-Aldrich, St Louis MO, USA).

### Experimental Design

One-day-old SPF White Leghorn chickens (*n* = 64) were randomly allocated to four groups with four replicates in each one (*n* = 4 chickens/replicate). The birds in group one did not receive the SC and served as an untreated–uninfected control group (UU). During the prepatent period of *E. tenella* infection, chickens in group two were individually treated once per day by oral gavage with 2 ml of SC (60 mg/bird), while chickens in group three were also individually treated once per day by oral gavage, but with 4 ml of SC (120 mg/bird). The birds in group four did not receive the SC and served as an untreated–infected control group (UI). At 86 days of age, all birds were weighed, and those in groups 2, 3, and 4 were orally (naturally) infected with 3 × 10^4^ sporulated oocysts of *E. tenella* each. The clinical signs and mortality of birds in every group were recorded daily. Feces from every group were collected separately at 5–7 days PI. The feed consumption during the prepatent period was recorded. At 7 days PI, all chickens were weighed, wing-bled, and euthanized in accordance with NOM 033-ZOO-2010 (Mexican regulation). Table 1 shows the details for the group and experimental design.

**Table 1:** Group and experimental design.

**Group**	**Number of chickens**	**Therapeutic supplement**	**Daily dosage (mg/bird)**	**Days of therapeutics**	**Age of infection (d)**	**Dose of oocysts per bird^**b**^**
1	16	None	None	0	Uninfected	None
2	16	SC IgY^a^	60	7	86	3 × 10^4^
3	16	SC IgY^a^	120	7	86	3 × 10^4^
4	16	None	None	0	86	3 × 10^4^

a *Drinking water suspension of egg yolk hyperimmune IgY antibodies against Eimeria sp. (Supracox®, SC; I.A.S.A., Puebla, Mexico)*.

b *Sporulated oocysts of a wild-type strain of E. tenella*.

### Parameters to Assess Immunotherapy Efficacy

The effectiveness of passive immunity was evaluated based on survival rate, body weight gain (BWG), lesion score, feed conversion ratio (FCR), range of hematocrit (HCT), oocyst output from the feces per gram (OPG), oocysts per gram of the ceca tissue (OPGC), and anticoccidial index (ACI). Survival rate was calculated dividing the number of surviving chickens × 100 by the number of initial chickens. The body weight (g) of every bird was measured at 86 days of age (before infection) and at 93 days (7 days PI). The relative BWG was calculated as follows: the average body weight gain of chickens in the treated group or untreated–infected control group × 100 / the average body weight gain of chicks in the untreated–uninfected control group. At 7 days PI, the cecal lesions from all infected birds were scored on a graded scale in a blinded fashion by two independent observers following the method described by Johnson and Reid (33). FCR was expressed as a ratio of grams of feed consumed to grams of body weight gained during prepatency. At 7 days PI, every chicken was wing-bled and the HCT ratio was determined for every blood sample. For OPG counting, chicks from every group (16 chicks/group) were placed in collection cages (4 chicks per collection cage) and fecal droppings were collected between 5 and 7 days PI. Pooled fecal material was suspended in 2 L of 2.5% potassium dichromate and OPG was determined according to the method of Long et al. (24). The oocysts per gram of cecal tissue (OPGC) were counted as previously described by Juárez et al. (25). ACI is a synthetic criterion for assessing the protective effect of an anticoccidial drug and is calculated as follows: (survival rate + percent relative weight gain) − (lesion index + oocyst index) (34).

### Statistical Analysis

All data were analyzed by ANOVA using the general linear model (GLM) of SAS (SAS/STAT 9.2 software, SAS Institute Inc., Cary, NC, USA). The normal distribution of residuals and variance homogeneity of the data were tested by UNIVARIATE procedure and the Bartlett's test, respectively. The chicken euthanized for observations (four for every replicate) of coccidiosis lesion scores and HCT ratios, constituted an experimental unit. These data were expressed as arithmetic means ± SD (standard deviations). For all the remaining traits studied, every cage was used as an experimental unit, and data were expressed as means ± SEM (standard errors of means). When the overall analysis showed the presence of significant differences between groups, significant differences (*P* < 0.05) between means were separated by a *post-hoc* analysis (Tukey's test).

## Results

### Antisera Against *E. tenella*

**Figure 1:**
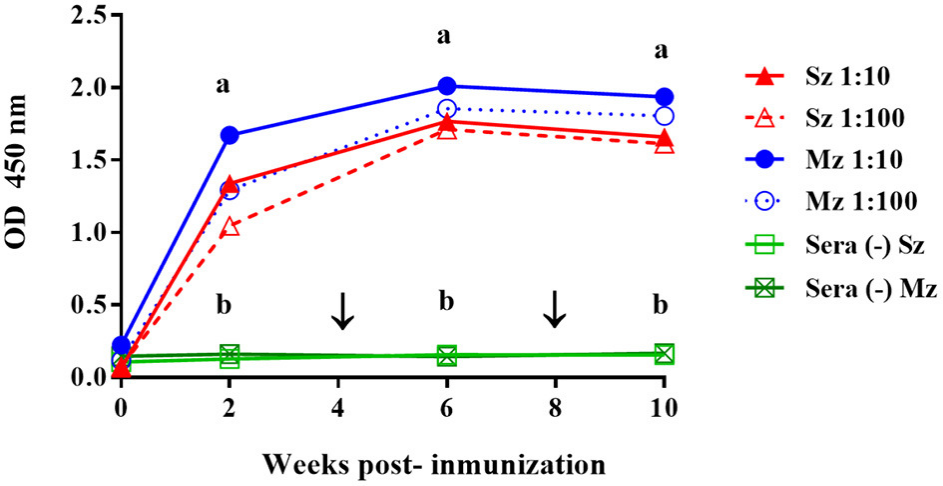
Comparison of the time course of the antibody response to sporozoite (Sz) and merozoite (Mz) antigens in pullets immunized with *E. tenella* by gavage. Sera dilutions of 1:10 (continuous line) and 1:100 (dotted line) are shown. The arrows indicate the boost immunizations at intervals of 4 weeks. Sera from unimmunized pullets (–) did not show any reactivity with the Sz and Mz antigens. At each time point, the arithmetic means ± standard deviations of the absorbance units (O.D.) of sera collected from six birds are shown. The line plots denoted as a are significantly different (*P* < 0.05, according to Tukey's multiple range test) from those denoted as b.

The kinetics of the antibody response to both asexual stages of *E. tenella* following immunization with 5 × 10^3^ sporulated oocysts of *E. tenella* in hybrid pullets increased over time, as shown by the ELISA (Figure 1). The antibody response to sporozoite and merozoite antigens was of similar intensity and followed the same pattern. From 2 weeks PI, both responses were different (*P* < 0.05) from those of unimmunized birds. For both antigens, high antibody levels were detected 2 weeks after the first immunization and peaked 2 weeks after the first boost immunization. The antibody levels then declined slightly toward 10 weeks PI, and the second booster did not show any effect on the antibody levels (Figure 1).

### Antisera Against Whole Sporozoites of *E. tenella*

**Figure 2:**
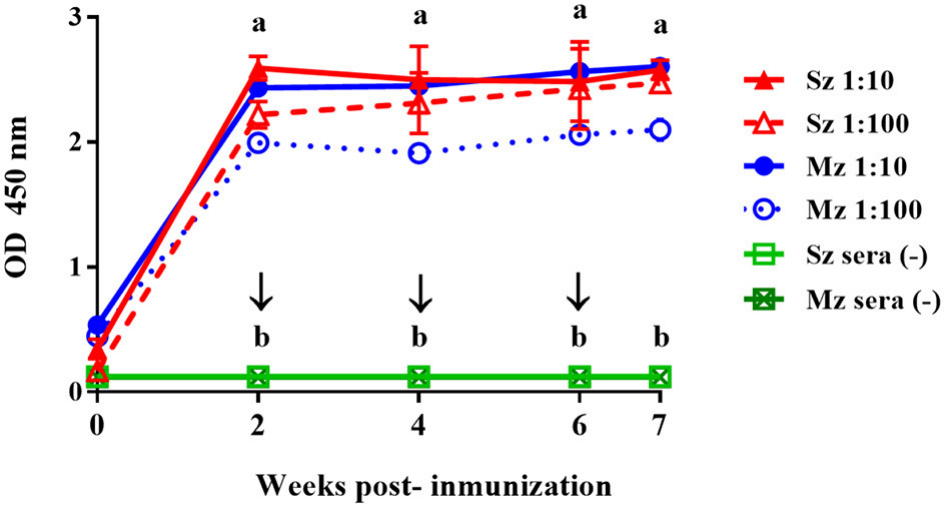
Antibody response to sporozoite (Sz) and merozoite (Mz) antigens in SPF Leghorn chickens immunized subcutaneously with whole sporozoites of *E. tenella*. Sera dilutions of 1:10 (continuous lines) and 1:100 (dotted lines) are shown. The arrows indicate the boost immunizations at intervals of 2 weeks. Sera from the four unimmunized birds (–) did not show any reactivity with Sz and Mz antigens. At each time point, the arithmetic means ± standard deviations of absorbance units (O.D.) of sera collected from four chickens are shown. The line plots denoted as a are significantly different (*P* < 0.05, according to Tukey's multiple range test) from those denoted as b.

The kinetics of the antibody response to sporozoite and merozoite antigens following immunization with the experimental vaccine to SPF White Leghorn chicken were compared (Figure 2). The antibody response to both asexual zoite-stage antigens was of similar intensity and followed the same pattern. For both antigens, high antibody levels peaked 2 weeks after first immunization; the antibody levels then plateaued until 6–7 weeks PI (Figure 2). From the sera peak until the end of the immunization schedule, antisera (1:100) reactivities against sporozoite antigens were slightly higher than that against merozoite antigens (Figure 2).

### SDS-PAGE of Egg Yolk IgYs From *Supracox®*

**Figure 3:**
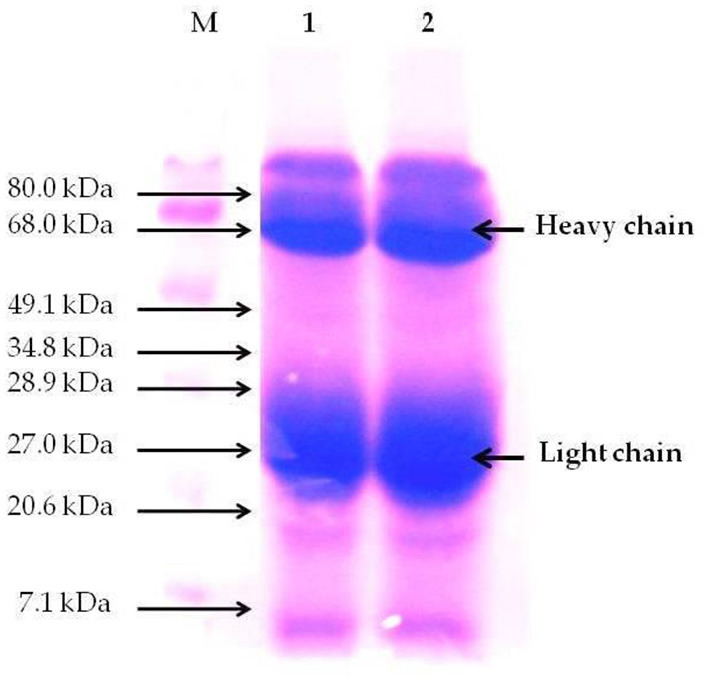
Sodium dodecylsulfate polyacrylamide gel electrophoresis (SDS-PAGE) pattern of purified egg yolk immunoglobulins (IgY) of Supracox® (SC). M stands for protein molecular weight marker; lane 1 and 2, IgY purified by spray-drying. The bold arrows indicate the heavy and the light chains of IgY.

The average yield of IgYs in the SC suspension was 30 mg/ml. The purity of IgY from the SC was up to 91%, as revealed by SDS-PAGE. The SC contains two major protein bands (lane 1 and 2) representing heavy chains and light chains of IgY, with expected molecular weights of ≈68 and ≈27 kDa, respectively (Figure 3).

### ELISA of Supracox® and Egg Yolk IgYs Against Whole *E. tenella* Sporozoites

**Figure 4:**
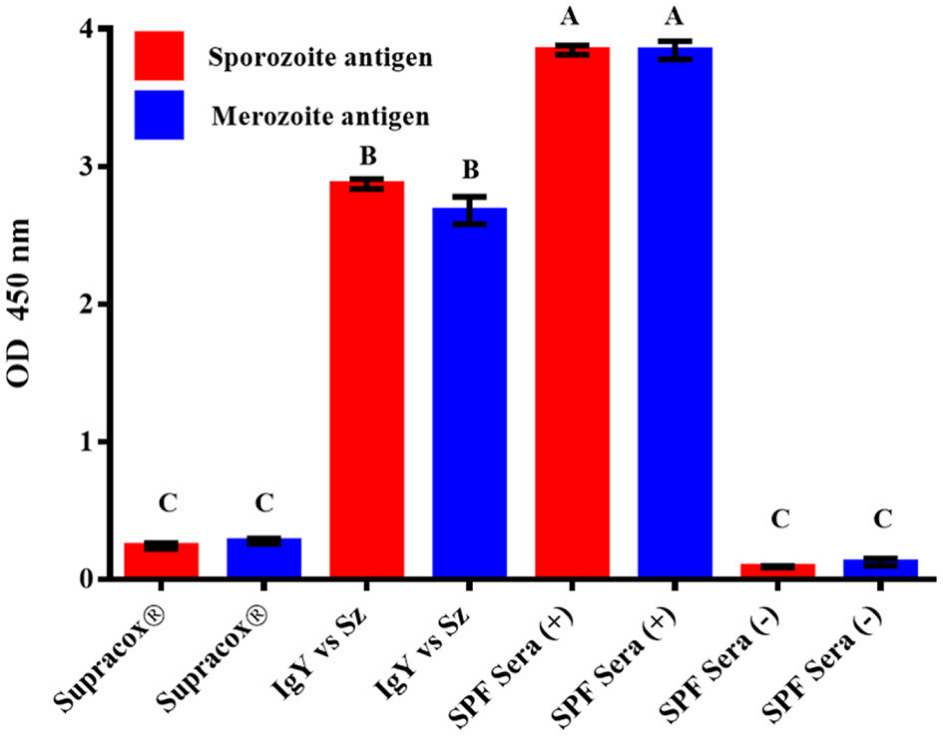
Reactivity of the IgYs in Supracox®, polyclonal egg yolk IgYs from birds immunized with the whole *E. tenella* sporozoites (IgY vs. Sz), positive antisera [SPF Sera (+)], and negative sera [SPF Sera (–)] to sporozoite (Sz) and merozoite (Mz) antigens in ELISA. Sera from unimmunized SPF White Leghorn birds did not show any reactivity to sporozoite and merozoite antigens. Each bar represents the arithmetic mean ± standard deviation of absorbance units (O.D.) (*n* = 6) of two independent observations (sera at 1:100). Bars that are not denoted by the same letter (A–C) represent significantly different values (*P* < 0.05) according to Tukey's multiple range test.

The immune reactivity of positive antisera from SPF White Leghorn chickens to merozoite and sporozoite antigens was the highest, and it was identical (3.84 O.D.) for both supernatants (Figure 4). The antibody titers from polyclonal egg yolk immunoglobulins against the whole *E. tenella* sporozoites showed a higher reactivity than the IgYs from the SC. The polyclonal egg yolk IgYs from birds immunized with the experimental vaccine showed a slightly higher reactivity against sporozoite (2.87 O.D.) than against merozoite antigens (2.68 O.D.). The SC showed low levels of antibodies, and the reactivity against merozoite antigens (0.28 O.D.) was slightly higher than the antibody response to sporozoite antigens (0.24 O.D.). The immunoreactivity of sera from unimmunized SPF White Leghorn chickens to both asexual zoite stages of *E. tenella* antigens was the lowest (<0.12 O.D.) (Figure 4).

### Western Blot of Supracox® and Egg Yolk IgYs Against Whole *E. tenella* Sporozoites

**Figure 5:**
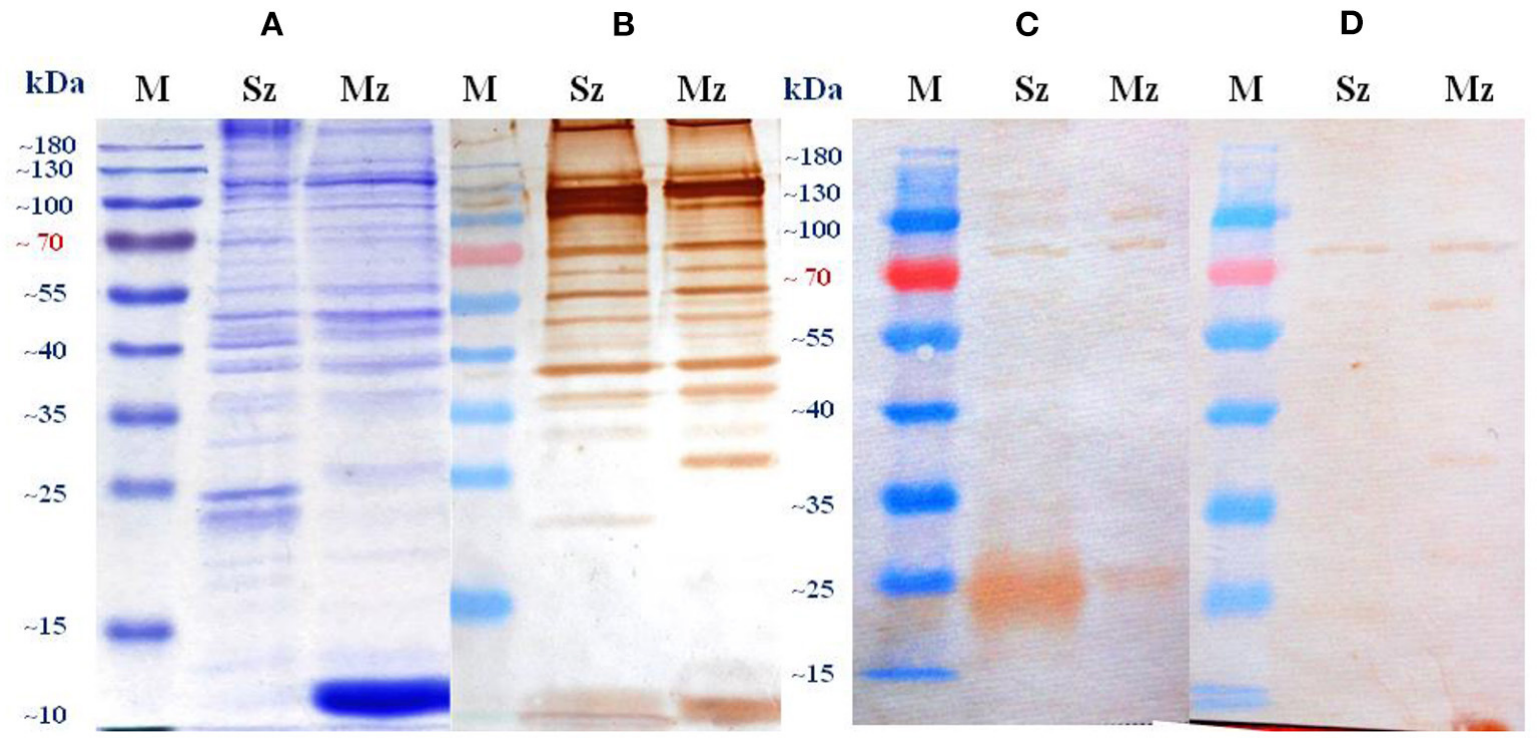
Sporozoite (Sz) and merozoite (Mz) proteins of a wild-type *E. tenella* strain resolved by SDS-PAGE on a 12% gel and stained by Coomassie brilliant blue **(A)** or probed in immunoblots with pooled antiserum from birds orally (naturally) immunized with sporulated oocysts of *E. tenella* **(B)**, polyclonal egg yolk IgYs from birds immunized with whole *E. tenella* sporozoites **(C)**, and polyclonal egg yolk IgYs from birds immunized against *Eimeria sp*. (Supracox®) **(D)**. Sera from unimmunized SPF White Leghorn birds did not show evidence of reactivity (data not shown). The pooled antiserum was diluted 1:100 in **(B)** and the egg yolk IgYs suspension was diluted 1:25 in **(C, D)**. M stands for protein standard.

The polypeptide profiles of sporozoites and merozoites in the CBB-stained gel were complex but similar between them with more than 25 bands identified in each stage. Some bands were common to both stages, while others were stage-specific (Figure 5A). Differences in the protein profiles of the two asexual zoite stages were particularly evident at molecular weights of <50 kDa. Sporozoites showed either single bands at about ≈230, ≈120, ≈105, ≈94, ≈68, ≈47, ≈42, and ≈38 kDa, as well as two bands between ≈22 and ≈25 kDa. Merozoites showed single bands at ≈120, ≈105, ≈56, ≈47, ≈44, ≈38, and ≈26, with a strongest stained large diffuse band in the ≈10–12-kDa range.

The preparation of SC and the IgYs collected from SPF White Leghorn birds immunized with the experimental vaccine were independently used to probe the resolved proteins of purified Sz and Mz antigens. Only a small proportion of the total proteins present on CBB-stained gels was recognized by the experimental IgYs (Figures 5C,D). For both asexual zoite stages of *E. tenella*, there were differences in the number and intensity of the staining of antigens recognized by each IgY preparation. The strongest reaction of polyclonal egg yolk IgYs from SPF birds inoculated with whole *E. tenella* sporozoites occurred with the Sz antigen. Sporozoite antigens single bands were identified at ≈120, ≈105, ≈82, ≈64, ≈47, and ≈17.5 kDa, as well as large diffuse band between ≈22 and ≈27 kDa, additionally, the two proteins at ≈34 and ≈27 kDa were barely visible in this blot (Figure 5C). This same preparation reacted with two common merozoite antigens at ≈105 and ≈82 kDa, as well as with an immunodominant merozoite-specific antigen in the ≈25–27 kDa range (Figure 5C). The SC showed a wider pattern of recognition in the merozoite than in the sporozoite (Figure 5D). The Western blot analysis showed that the SC reacted clearly with an ≈82 kDa protein and very weakly with four proteins of ≈60, ≈34, ≈24, and ≈17 kDa in the sporozoite. The clearest protein bands detected with the SC in the merozoite were at ≈82, ≈60, ≈54, ≈40, ≈38, ≈27.5, and ≈13 kDa, and three proteins were detected less clearly at ≈24, ≈20, and ≈17.5 kDa (Figure 5D). A dominant protein at ≈82 kDa was recognized in both asexual zoite stages of *E. tenella* by each egg yolk polyclonal IgY preparation. The antisera obtained from birds infected orally (naturally) with sporulated oocysts of *E. tenella* recognized 13 intensely stained protein bands in the sporozoite antigen and 14 immunodominant bands in the merozoite antigen (Figure 5B). Several of these protein bands were also recognized by both egg yolk polyclonal IgYs preparations, but with less intensity.

### Parameters of Protection Offered by Supracox®

**Figure 6:**
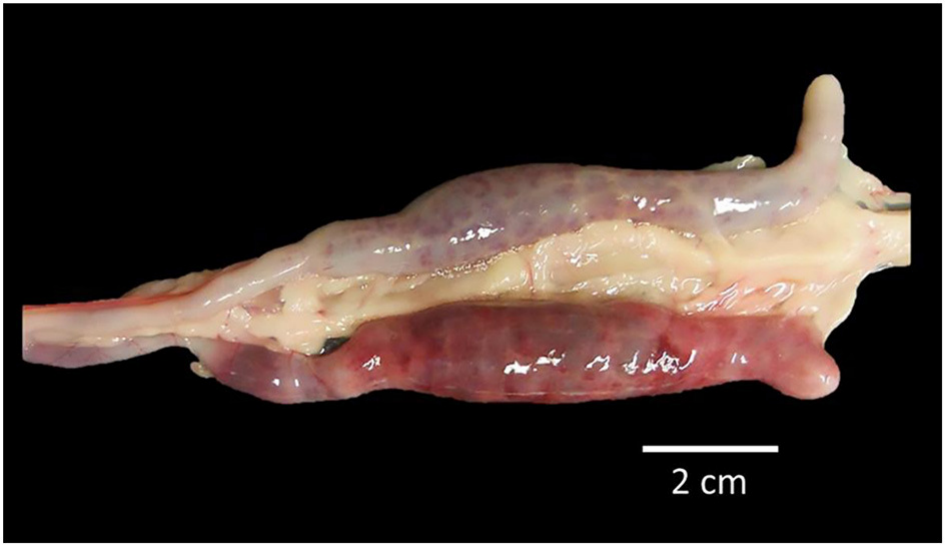
Severe lesions in both ceca sacs of SPF White Leghorn chick infected with a high dose of sporulated oocysts of *E. tenella* and treated by oral gavage with purified egg yolk immunoglobulins (60 mg IgY) of Supracox® during prepatent period, all birds from this group recovered quickly after the patency period.

**Figure 7:**
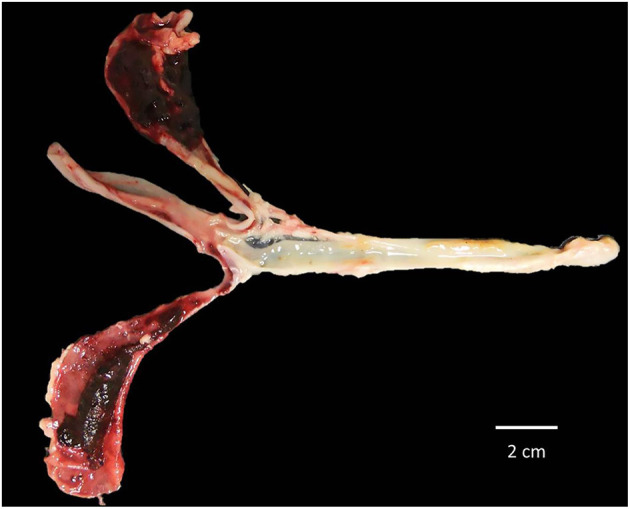
Severe lesions in ceca sacs of SPF White Leghorn chick infected with a high dose of sporulated oocysts of *E. tenella*, this bird did not receive any treatment of immunotherapy based in purified egg yolk immunoglobulins (IgY) of Supracox® during prepatent period, at least 25% of these birds died through this time.

The protective efficacy of the SC against a severe *E. tenella* infection in terms of BWG, FCR, HCT, survival rate, mean lesion score, OPG, OPGC, and ACI is shown in Tables 2, 3. Although chickens in the untreated-infected group showed a 25% mortality and none of the SPF Leghorn chickens supplemented with 60 mg of SC died, the remaining traits studied did not show any differences between these two groups. The birds supplemented with 120 mg of SC and challenged with a large dose of *E. tenella* were successfully immunoprotected. The results from this group were statistically indistinguishable from the results observed in the UU control group. The BWG of the group treated with 60 mg of SC was significantly lower (*P* < 0.05) than the BWG of the UU control group and the group treated with 120 mg of SC (Table 2). The group treated with 60 mg of SC showed the highest feed conversion ratio. The HCT was normal for chickens in the group treated with 120 mg of SC and in the UU group. The HCTs of chicks with 60 mg of SC and birds of the UI control group were significantly lower (*P* < 0.05) than normal HCT (Table 2). A significant alleviation (*P* < 0.05) of the cecal lesion was observed in birds from the group treated with 120 mg of SC compared with the cecal lesion score recorded in the 60 mg of SC and UI groups (Figures 6, 7). No statistically significant differences were shown in the lesion score between the 60 mg SC group and the UI group (Table 3). The highest reduction in OPG was observed in the 120 mg SC group compared with the OPG from the 60 mg SC group or the UI control group. In the 120 mg SC group, the reduction in OPG was up to 71.8% (Table 3). Compared with the 60 mg SC group, the birds treated with 120 mg of SC showed a significant reduction in OPGC after infection with *E. tenella*. In the infected groups, the ACI of the untreated–infected group was the lowest (47), and the group treated with 120 mg of SC showed the highest ACI (155) (Table 3).

**Table 2:** Parameters of protection at 7 days post-infection with a crowded dose of *E. tenella* to SPF White Leghorn chickens supplemented with *Eimeria* sp.-specific IgYs during the prepatent period.

**Group**	**Average body weight (g)^**1**^**	**Relative body weight gain (%)**	**Feed conversion ratio (g:g)^**1**^**	**Hematocrit (%)^**1**^**	**Survival rate (%)**
Uninfected control	963 ± 7.51^a^	100	6.03 ± 0.13^c^	30.8 ± 1.45^a^	100
IgY (60 mg)	860 ± 90.0^b^	89.3	48.44 ± 35.7^a^	27.7 ± 7.1^b^	100
IgY (120 mg)	918 ± 13.1^a^	95.3	6.73 ± 0.31^c^	34.9 ± 3.7^a^	100
Infected control	891 ± 60.0^ab^	92.5	23.56 ± 18.4^b^	27.4 ± 4.6^b^	75

1 *Data within columns with different letter superscripts (a–c) are significantly different from each other (P < 0.05)*.

**Table 3:** Immunotherapy protection by the *Eimeria* sp.-specific IgYs against a severe *E. tenella* infection in SPF White Leghorn chickens.

**Group**	**Mean lesion score^**1**^**	**Oocysts per fecal gram^**1**^ (10^**6**^)**	**Oocysts per cecal gram^**1**^ (10^**6**^)**	**Anticoccidial Index (ACI)**
Uninfected control	0.0 ± 0.0^c^^1^	0.00^c^	0.00^c^	200
IgY (60 mg)	3.25 ± 0.86^ab^	0.31 ± 0.21^a^	2.13 ± 1.53^a^	55
IgY (120 mg)	2.5 ± 0.67^b^	0.11 ± 0.079^b^	1.22 ± 0.82^b^	155
Infected control	3.40 ± 0.79^a^	0.39 ± 0.30^a^	0.92 ± 0.34^b^	47

1 *Data within columns with different letter superscripts (a-c) are significantly different from each other (P < 0.05)*.

## Discussion

Vaccination with live or attenuated oocysts of *Eimeria* sp. is routinely used to confer protection by inducing adaptive immunity. However, very little research has been conducted on the protection of poultry from clinical coccidiosis by passive immunization using parasite-specific hyperimmune egg yolk antibodies (18). Cellular and humoral immunity are both involved in the protection of chickens against *Eimeria* infection (1). Most studies have shown that cellular immunity is very important, whereas the role of humoral immunity is relatively minor (1, 20, 35). However, several studies have confirmed the ability of antibodies to block the invasion, development, and transmission of some pathogens in the poultry and livestock (6, 18). The oral administration of specific IgYs has been shown effective against gastrointestinal pathogens, such as *Escherichia coli, Campylobacter* sp., *Salmonella* sp., infectious bursal disease virus, Newcastle disease virus, rotavirus, and *Helicobacter pylori* (6, 18, 19, 36, 37). Passive and maternal immunity mediated by antibodies have been shown effective against *Eimeria* infection (8–11, 21–23, 38). However, since the early studies of Danforth and McAndrew (14) and Smith et al. (15) concerning immune protection with specific IgY against *Eimeria* sp., little attention has been paid to passive immune therapy for the control of clinical coccidiosis outbreaks in chickens.

The present study shows a significant anticoccidial therapeutic effect in birds treated with IgYs by oral gavage during an *E. tenella* clinical infection assay. The treatment of chicks challenged with a large dose of the wild-type strain of *E. tenella* with the IgY preparation SC resulted in null mortality, less oocyst shedding, normal hematocrit levels, lower cecal lesion score, and increased ACI, as well as an improvement in the growth performance and welfare status of the chickens. The infection dose of oocysts used in our study was higher than the quantity of oocysts used in similar studies (11, 21–23). A crowded dose was used in order to truly test the therapeutic effect of supplementation with these specific IgY *Eimeria* sp. (SC) in conditions closest to the worst-case scenario of an unexpected *Eimeria* infection occurring in a broiler barn when birds are growing (39). As a matter of fact, our data indicated that the dose given as a therapeutic prescription from the manufacturer of this IgY suspension (60 mg/bird/daily) prevented deaths in chickens in this group, but this dose did not protect as successfully against clinical signs as the higher dose of IgYs we used here (120 mg/bird/daily) (40). The passive immune protection offered by the lower dose of IgY treatment was weak, while successful immune protection with a higher dose of IgYs was maintained throughout prepatent period. These differences were probably due to the differing efficiencies of the two IgY quantities by themselves. Hence, the partial protection by the lower dose of IgYs could have been due to the higher quantity of sporulated oocysts present in the crowded dose used in our *E. tenella* infection trial (39). Apparently, the quantity of oocysts overcome the minimum amount of IgYs that is required to neutralize the *E. tenella* infection. Indeed, it was much higher than the quantity of oocysts that broilers normally are exposed to under field conditions (8). Nevertheless, none of the birds experimentally infected and treated with any dose of SC died; instead, they recovered quickly from the illness, while 25% of the birds that were infected and did not receive any treatment died.

To the best of our knowledge, the preparation of egg yolk-specific IgYs against *Eimeria* sp. has never been used before in any experimental assay to evaluate the immune therapeutic effect on clinical coccidiosis experimentally induced with a large dose of *E. tenella*. At first glance, we can conclude that the dose of IgYs that the manufacturer of the SC recommends (60 mg of IgY/bird/day) (40) might be used in the field as a valuable immune therapeutic tool against *E. tenella* outbreaks; however, in order to avoid any negative effect on the future performance of flocks, we recommend applying the SC at 120 mg of IgY/bird/day. Additionally, it should be considered that dosage lower than 60 mg of IgY/bird/day could put down birds at greater risk of death.

The recovery of fewer sporozoites of *Eimeria tenella* from the lumen of the ceca of homologously challenged immunized chickens suggests that the intraluminal destruction of the initial invasive stages may be one of the main mechanisms involved in the immune-mediated restriction of the replication of *Eimeria* (41). The most likely explanation of this effect is the action of secreted specific antibodies (42). Such antibodies have been detected in the gut, and their concentration is directly correlated with the magnitude of the effect (41, 42). While the exact mechanism through which IgY counteracts pathogen activity has not been determined, it is known that neutralization mechanisms largely depend upon efficient antigen–antibody interaction (19). Some researchers have proposed several mechanisms to explain how the specific IgYs protect against pathogen activity (6, 18). The main one involves binding of the antibodies to specific antigens on the pathogens, thus impairing their growth or biological functions. For example, the IgY antibody binds to bacterial structures, such as the flagella, fimbriae (sexual pili), outer membrane proteins, and lipopolysaccharide (LPS), and inhibits bacterial adhesion to the intestinal wall. This reduction in adherence to the lining reduces bacterial growth and their ability to colonize the intestinal cells (18, 43). Tsubokura et al. (36) suggested that the inhibition of bacterial growth or colonization observed due to IgYs treatment could be the result of bacterial agglutination, causing a reduction in colony forming units, rather than of direct effects on individual bacteria. Although agglutination may be a mediator of growth inhibition, it is unlikely to be the most important one because cross-linking of bacteria is precluded by the steric hindrance of the Fab arms of the IgYs bound to their surface (43). Although Rose and Hesketh (41) speculated that agglutination could be the major inhibitory mechanism in the invasion of *E. tenella* sporozoite in the enterocytes of immunized hosts, several *in vitro* studies have suggested that inhibition of adhesion is the dominant mechanism by which specific IgY counteracts *Eimeria* activity (14, 16, 38). Opsonization followed by phagocytosis, inhibition of enzyme activity, and toxin neutralization are other mechanisms proposed to explain the action of IgY (18, 19). Binding of specific IgY to bacteria may also alter the bacterial cellular signaling cascades and thus reduce the production and release of toxins (18). Although the IgYs applied against *Eimeria* sp. in our study could prevent the internalization of both asexual and sexual zoite stages of *E. tenella* in enterocytes, suggesting adherence-blockade or deactivation of *Eimeria*-specific invasion proteins (e.g., AMA1–RON2 interaction) by the inhibition of its enzyme activity or proteolytic processing (18, 43, 44), the exact mechanism through which these specific IgYs protected birds is not yet known. Knowing the major mechanism of action of these IgYs could contribute to the better design of strategies and specific immunoglobulins to obtain the most efficient immune protection effects. Therefore, further intensive studies are needed to uncover the major anti-protozoal action of IgYs against *Eimeria* sp. infections.

In the present study, the BWG, oocyst shedding, viability, hematocrit, lesion score, and ACI were used for the accurate assessment of the immune protection by IgYs. When all parameters were analyzed separately or using the ACI only, protective effects were observed in the group treated with a higher dosage of IgYs. This desirable result might be due to the targeting of the polyclonal IgYs against many different life-cycle stages of *E. tenella*. However, which of these stages are more affected is not yet known. Interestingly, while in the immunoblots, the SC preparation showed the most reactivity to merozoite antigens, the egg yolk IgY preparation from SPF birds immunized with the experimental vaccine was more reactive to the sporozoite antigen. Further studies are needed to elucidate which specific life-cycle stage of *Eimeria* sp. is more affected by each experimental polyclonal IgY (10, 11, 29). When the results of the group supplemented with 120 mg of SC were compared with the outcome of the untreated–infected control group, in terms of all studied traits, the IgYs clearly showed an enhanced resistance of birds against the *E. tenella* infection. However, when birds supplemented with 60 mg of SC were compared with this UI group, no significant difference in BWG, hematocrits, oocyst output, or cecal lesion score were observed, while the only significant difference observed was in terms of mortality. The differences observed between both groups treated with the SC might be associated with the antibody titer levels of IgYs reached at the intestinal level of every bird (41, 42). This would suggest that the quantity of IgYs taken orally by every bird could show a dose–response relationship, as discussed below.

Although the group supplemented with 60 mg of the SC only differed compared to untreated–infected group in terms of viability; the reason why none of the birds died in this group is not known. Some researchers have pointed out that when germ-free chicks (gnotobiotic) were challenged with large doses of pathogenic sporulated oocysts of *E. tenella*, although some birds showed severe lesions and profuse bleeding into the ceca, none died (3, 4, 45, 46). However, when birds with a “normal” gut microbiota were challenged even with less *E. tenella* oocysts than dose used to challenge of gnotobiotic birds, almost all the “normal” birds displayed severe cecal lesions, and, unexpectedly, some of them died without exhibiting clinical signs (3, 4, 45, 47). According to Kimura et al. (46), serious infection with *Eimeria* sp. increases the growth of pathogenic microorganisms, such as *Clostridium perfringens*, during the first 7–10 days PI; this puts gut microbiota out of balance during the 2 or 3 weeks after infection with *Eimeria* sp. In this same period, Macdonald et al. (3) observed that all differential operational taxonomic units (OTUs) belonging to the family Enterobacteriaceae showed a moderate but persistent increase. MacDonald et al. (3) later showed that the main pathology effect in the ceca after enterocyte damage by *E. tenella* infection can in great part be attributed to microbiome dysbiosis caused by anaerobic and facultative-anaerobic organisms, such as coliform and non-coliform Enterobacteriaceae and *Clostridium* sp. Hence, the pathological damage caused by *E. tenella* infection is strongly associated with cecal microbiota dysbiosis (4).

Although the birds in the group treated with 60 mg of SC did not display a successful antiprotozoal protection, as shown by the group supplemented with 120 mg of SC, none of these birds died. According to the SC blot analysis, some IgYs recognized several specific epitopes in the *E. tenella* sporozoites and several more in the merozoites; we cannot be sure that all the IgYs in the SC are involved in conferring 100% immune protection against *E. tenella* infection by themselves. Therefore, we can deduce that after *E. tenella* infection, the SC might have other IgYs (e.g., vs. *E. acervulina* and *E. maxima*) or undefined substances that, by unknown mechanisms, counteract the pathogen activity of *E. tenella* by itself or from secondary opportunistic bacteria, thus preventing the deaths of these birds. An examination of the immune protection mechanism that these unknown IgYs or substances display upon bacterial interaction at the gut level while a pathogenic *Eimeria* sp. infection occurs might be illuminating.

In order to evaluate their prophylactic action, in previous studies, IgY powder from the egg yolks of layer hens immunized with *Eimeria* sp. was orally supplemented to chickens during the growing period. In contrast, the present study analyzed the therapeutic action of the hyperimmune egg yolk *Eimeria* sp.-specific IgY suspension supplemented by oral gavage during a severe *E. tenella* infection. The therapeutic dosage of IgYs supplemented here significantly affected the protection efficacy in an apparent dose–response relationship. Similar results with a dose–response feature were also seen with similar lyophilized products supplemented as prophylactics in feed (21–23). Lee et al. (21) reported that the supplementation of broiler diets with low levels of hyperimmune IgY egg yolk powder (SC) (0.01, 0.02, 0.05, or 0.5%) significantly reduced oocyst production but had no effect on the BWG when the chickens were challenged with 1 × 10^4^ oocysts of *E. acervulina*, while supplementation with higher levels of SC (10 or 20%) protected the birds from subsequent challenge with *E. acervulina*, and the birds showed greater BWG and lower oocyst production than observed in the untreated–challenge group. Lee et al. (22) also showed similar effects, with increased BWG, reduced intestinal lesions, and less fecal oocyst shedding following challenge with either *E. maxima* or *E. tenella* when birds were fed diets supplemented with SC in a dose–response relationship. In both reports, hyperimmune IgYs were produced in laying hens immunized with the three major species of *Eimeria* (*E. acervulina, E. maxima*, and *E. tenella*) (21, 22). In another study, hyperimmune IgY antibodies were generated, immunizing layer hens with five species of *Eimeria* (*E. acervulina, E. maxima, E. tenella, E. necatrix*, and *E. praecox*), and the IgYs were isolated in pure form and lyophilized (23). When birds were fed with this lyophilized powder at different levels (0.01, 0.02, 0.05, 0.10, 0.50, or 1%), this IgYs compound conferred protection against challenge with 1 × 10^4^ oocysts of *E*. *tenella* in a dose–response relationship. Xu et al. (23) supplemented a group of birds with the highest level of lyophilized powder (1.0 %); subsequently, the group was challenged with 1 × 10^4^ oocysts of *E tenella*, showing an ACI of 160.4. Although this value was slightly higher than the ACI we recorded here for our group treated with 120 mg/day/bird of hyperimmune *Eimeria* sp.-specific IgYs (SC) (ACI = 155), we have to consider that the *E. tenella* infection dose in our group was higher (3 × 10^4^ sporulated oocysts) than the infection dosage administered by Xu et al. (23). This indicates that the SC supplemented as immunotherapy at the higher dose had as successful a protection efficacy as the lyophilized powder tested by Xu et al. (23) at their highest level. All these studies strongly suggest that supplementing diets or drinking water with hyperimmune egg yolk *Eimeria* sp.-specific IgYs is a promising strategy for controlling unexpected infections of avian coccidiosis, and might significantly reduce the economic losses caused by this disease.

Thus far, all these studies have used live parasites administered through the oral (natural) route in their hyperimmunization programs. Our Western blot results showed that several bands were clearly identified on both life-cycle stages of *E. tenella* from pullets that were orally (naturally) immunized, while few antigen bands from both asexual zoite stages of *E. tenella* were recognized by the SC preparation. On the other hand, the ELISA reactivity of the SC preparation toward both asexual zoite stages of *E. tenella* was lower compared with the reactivity shown by the egg yolk IgYs preparation from SPF birds immunized subcutaneously with whole sporozoites of *E. tenella*. The SC preparation apparently showed a lower reactivity in both ELISA and Western blot analyses; regardless of this, the IgYs in the SC successfully protected the birds against large infection with *E. tenella*. Although we found that SPF Leghorn chickens immunized subcutaneously with intact sporozoites produce antibodies that apparently recognized the same specific antigens at each stage, this antisera showed a slight preference toward sporozoite antigen. SPF White Leghorn chickens injected with the complete sporozoite vaccine apparently show a higher level of humoral response (Sz and Mz = ≈2.6 OD) than pullets immunized by natural (oral) route (Sz and Mz = ≈1.9 OD) (Figures 1, 2). This is consistent with previous evidence that showed that the sporozoites parentally inoculated are more immunogenic by this route than birds naturally (orally) immunized (48). A parenteral route of immunization would be a suitable way to obtain a high amount of circulating specific-antibodies and, consequently, enhance the titers of *Eimeria* sp.-specific IgYs in egg yolk (10, 11, 20, 26). Furthermore, the accurate identification of immunogenic parasite proteins targeted by a protective antibody response against avian coccidiosis will allow the application of passive immunization technology (10, 11, 29, 32, 38, 49). Further studies concerning immunization strategies using defined protective *Eimeria* antigens or their epitopes injected through the parenteral route are required. Chicken monoclonal antibodies against parasite molecules involved in cell invasion have already been described (8, 14, 38, 49). The construction of a phagemid display antibody library of a single-chain variable fragment (scFv) that can block *Eimeria* invasion is another promising technology that could lead to the development of a novel immunotherapy strategy against coccidiosis (16, 17, 50). Such technologies must be developed to identify the key protective epitopes of *Eimeria* parasites, and further research on this issue is warranted. Oral immunotherapy represents a novel strategy to prevent severe pathological lesions and mortality when a large dose of *E. tenella* unexpectedly infects birds and the use of anticoccidials, such as chemical drugs or ionophores, is forbidden.

## Conclusions

The oral passive immunotherapy of chickens using *Eimeria* sp.-specific hyperimmune IgYs from egg yolk is powerful enough to control clinical signs, mortality, economic losses, and the pressure of infection by the parasite. Moreover, immunotherapy administered by oral gavage with egg yolk *Eimeria* sp.-specific IgYs represents a natural means to successfully control *E. tenella* infections.

## Data Availability Statement

The original contributions presented in the study are included in the article/supplementary material, further inquiries can be directed to the corresponding author.

## Ethics Statement

The animal study was reviewed and approved by Institutional Review Board for Husbandry and Care in Animals (CICUA) of the FMVZ-UNAM through the PhD internal board.

## Author Contributions

MJ-E: conceptualization, methodology, software, and writing—original draft preparation. MJ-E, FS-G, and RA-M: visualization and investigation. MJ-E and GT-I: data curation and statistical analysis. MJ-E, GT-I, and RA-M: funding acquisition, reviewing, and editing. RA-M: supervision. All authors contributed to the article and approved the submitted version.

## Funding

The research was supported in part by funds provided by USDA-NIFA Sustainable Agriculture Systems, Grant No. 2019-69012-29905. Title of Project: Empowering U.S. Broiler Production for Transformation and Sustainability USDA-NIFA (Sustainable Agriculture Systems): No. 2019-69012-29905.

## Conflict of Interest

The authors declare that the research was conducted in the absence of any commercial or financial relationships that could be construed as a potential conflict of interest.

## Publisher's Note

All claims expressed in this article are solely those of the authors and do not necessarily represent those of their affiliated organizations, or those of the publisher, the editors and the reviewers. Any product that may be evaluated in this article, or claim that may be made by its manufacturer, is not guaranteed or endorsed by the publisher.
